# Honokiol alleviates LPS-induced acute lung injury by inhibiting NLRP3 inflammasome-mediated pyroptosis via Nrf2 activation in vitro* and *in vivo

**DOI:** 10.1186/s13020-021-00541-z

**Published:** 2021-11-29

**Authors:** Yuhan Liu, Jiabin Zhou, Yingying Luo, Jinxiao Li, Luorui Shang, Fangyuan Zhou, Shenglan Yang

**Affiliations:** 1 Department of Integrated Traditional Chinese and Western Medicine, Union Hospital, Tongji Medical College, Huazhong University of Science and Technology, Wuhan, 430022 China; 2 Department of Neurosurgery, Union Hospital, Tongji Medical College, Huazhong University of Science and Technology, Wuhan, 430022 China; 3 School of Clinical Medical, Hubei University of Chinese Medicine, Wuhan, 430060 China

**Keywords:** Acute lung injury, Honokiol, NLRP3 inflammasome, Pyroptosis, Nrf2, Lipopolysaccharide

## Abstract

**Background:**

Honokiol (HKL) has been reported to ameliorate lipopolysaccharide (LPS)-induced acute lung injury (ALI). However, its potential mechanism of its protective effects remains unclear. In this study, the protective mechanism of HKL on LPS-induced ALI was explored in vivo and in vitro*.*

**Methods:**

In vivo, the SD rats were intratracheally instilled with LPS (5 mg/kg) to establish an acute lung injury model and then treated with HKL (1.25/2.5/5 mg/kg) or ML385 (30 mg/kg) intraperitoneally. In vitro, the human bronchial epithelial cell line (BEAS-2B) was stimulated with LPS and ATP to induce pyroptosis and treated with HKL (12.5/25/50 μM). Small interfering RNA (siRNA) technique was used to knockdown Nrf2 in BEAS-2B cells. The protein and mRNA expression levels of Nrf2, HO-1, NLRP3, ASC, CASP1, and GSDMD in cells and lung tissues were detected by western blot and real time-PCR. The expression levels of interleukin (IL)-1β, IL-18, MPO, MDA, and SOD in bronchoalveolar lavage fluid (BALF) and supernatant were determined by ELISA. The degree of pathological injury of lung tissue was evaluated by H&E staining.

**Results:**

The results showed that HKL could alleviate oxidative stress and inflammatory responses by regulating the levels of MPO, MDA, SOD, IL-1β, IL-18 in supernatant. And it could also inhibit the expression levels of NLRP3, ASC, CASP1, GSDMD via activation of Nrf2 in BEAS-2B cells. Further studies revealed that HKL could attenuate the pathological injury in LPS-induced ALI rats, and the molecular mechanism was consistent with the results in vitro.

**Conclusions:**

Our study demonstrated that HKL could alleviate LPS-induced ALI by reducing the oxidative stress and inhibiting NLRP3 inflammasome-mediated pyroptosis, which was partly dependent on the Nrf2 activation.

**Graphical Abstract:**

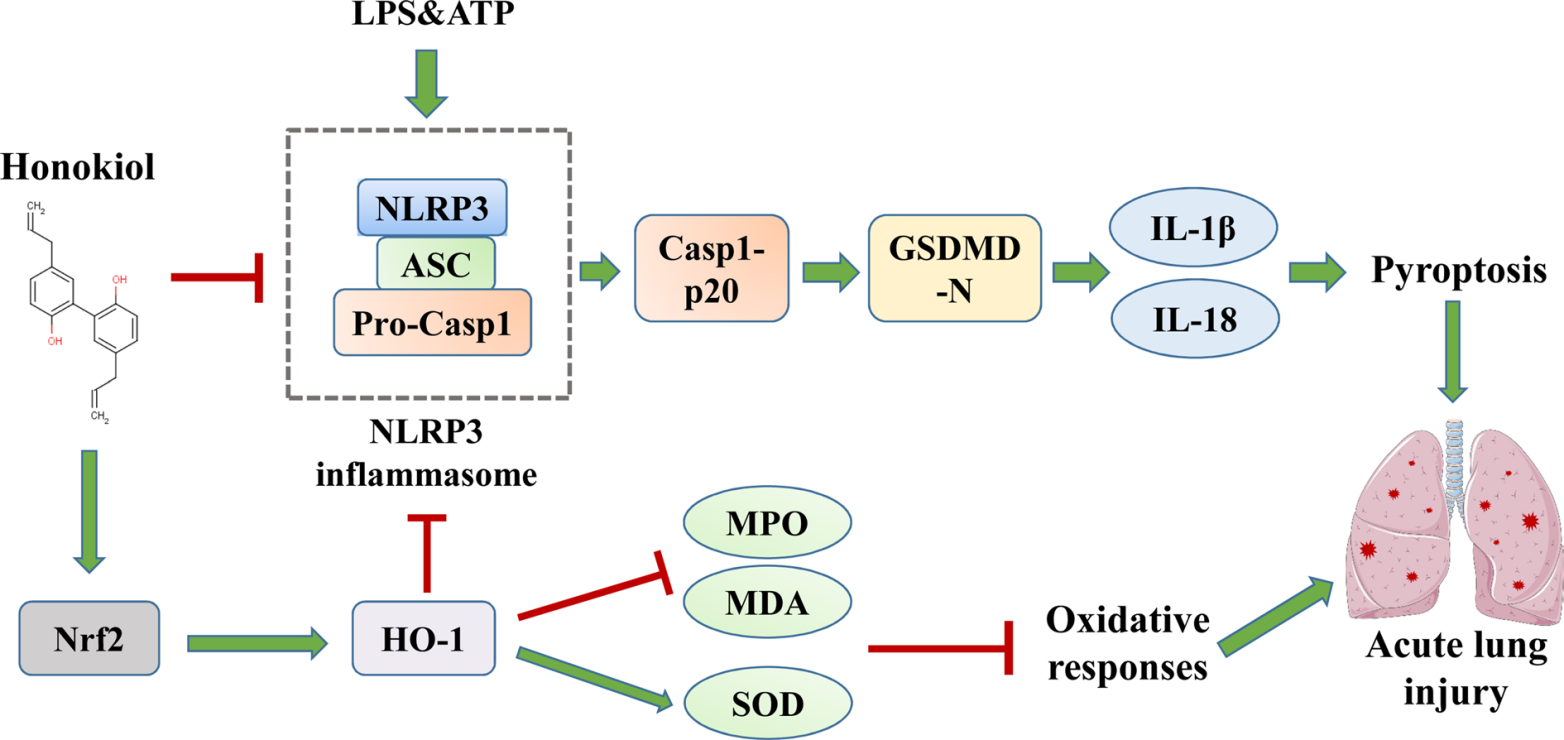

## Introduction

Acute lung injury (ALI) and acute respiratory distress syndrome (ARDS) are mainly responsible for hypoxic respiratory failure in adults with high morbidity and mortality during hospitalization [1]. ALI is characterized by the diffused lung inflammation, damage of lung epithelial barrier, and pulmonary edema [2]. Increasing evidence depicts that airway epithelial cells are involved in the pathogenesis of ALI/ARDS. As the first line of lung defense against lung injury, airway epithelial cells can produce the epithelial-associated inflammatory factors and participate in promoting oxidative stress, local response, and tissue damage [3, 4]. Therefore, airway epithelial cells are the potential therapeutic targets for preventing and treating ALI/ARDS.

The NOD-like receptor family pyrin domain containing 3 (NLRP3) inflammasome, is a vital component of innate immunity, comprising NLRP3, pro-caspase-1 (casp1), and apoptosis-associated speck-like protein containing a caspase-recruitment domain (ASC) [5]. The NLRP3 inflammasome activation requires two signals [6]: one being the transcriptional priming such as LPS and another one being ATP, nigericin, or monosodium urate (MSU) crystals. NLRP3 upon activation, processes the pro-casp1 into mature casp1, activating gasdermin D (GSDMD) that form holes in the cell membrane. As a result, the cells undergo pyroptosis releasing a large number of inflammatory factors such as IL-18 and IL-1β, and triggering severe inflammatory responses [7]. Recent evidence suggests that NLRP3 inflammasome-mediated pyroptosis in macrophages aggravates lung inflammation in LPS-induced ALI [8]. However, the role of pyroptosis in airway epithelial cells for the LPS-induced ALI remains unclear.

The transcription factor nuclear factor erythroid-2 related factor 2 (Nrf2) participates in regulating oxidative stress and inflammation [9, 10]. Nrf2 under oxidative stress is translocated into the nucleus and binding with anti-oxidant response elements (AREs) in the promoter region of the cell-protective genes, such as heme oxygenase-1 (HO-1) and NAD(P)H quinone dehydrogenase 1 (NQO1) [11]. Recent studies have shown Nrf2 could inhibit the activation of the NLRP3 inflammasome [12]. Nevertheless, there have been no reports stating whether Nrf2 can affect the NLRP3 inflammasome-mediated pyroptosis in the airway epithelial cells.

Honokiol (HKL), a natural compound extracted from *Magnolia officinalis*, possesses anti-inflammatory and anti-oxidant properties [13, 14]. Related studies highlight that HKL can activate Nrf2 to improve oxidative stress-induced tissue damage [15] and antagonize NLRP3, thereby reducing the inflammatory response [16]. But, there is no clear indication of whether HKL can inhibit NLRP3 inflammasome-mediated pyroptosis in LPS-induced ALI.

This study hypothesized that HKL can suppress NLRP3 inflammasome-mediated pyroptosis and reduce the oxidative stress by activating Nrf2, thereby attenuating LPS-induced ALI. Based on this hypothesis, a series of studies were carried out.

## Materials and methods

### Reagents

Honokiol (Catalog No: B20498) was purchased from Yuanye Bio-Technology Co (Shanghai, China). LPS (Catalog No: Escherichia coli 055:B5) was purchased from Sigma (St. Louis, MO, USA). ATP (Catalog No: A9310) was purchased from Solarbio (Beijing, China). ChamQ™ SYBR® qPCR Master Mix (Catalog No: Q311-02) and HiScript® II Q RT SuperMix for qPCR (Catalog No: R222-01) were obtained from Vazyme Biotechnology (Nanjing, China). Cell counting kit-8 kit was purchased from Dojindo (Kumamoto, Japan). Fetal bovine serum (FBS) was obtained from GIBCO (Grand Island, NY, USA) and Dulbecco’s modified Eagle’s medium/high glucose (DMEM, SH30022.01) and phosphate-buffered saline (PBS, SH30256.01) was purchased from HyClone (Logan, UT, United States). Hoechst 33342 (C1027) and Propidium Iodide (PI, ST511) staining solution were purchased from Beyotime Biotechnology (Shanghai, China). The IL-1β (Catalog No: E-EL-R0012c) ELISA kit and IL-18 (Catalog No.: SEA064Ra) for Rattus norvegicus were obtained from Elabscience Biotechnology (Wuhan, China) and USCN (Wuhan, China). The IL-1β (Catalog No: EK0392) ELISA kit and IL-18 (Catalog No: EK0864) for Homo sapiens were obtained from Boster Biological Technology (Wuhan, China). The myeloperoxidase (MPO) assay kit (Catalog No: A044-1-1), malondialdehyde (MDA) assay kit (Catalog No: A003-1-2) and Superoxide Dismutase (SOD) assay kit (Catalog No: A001-3-2) were obtained from the Jiancheng Bioengineering Institute of Nanjing (Nanjing, Jiangsu, China).

### Cell culture and treatment

The human bronchial epithelial cell line (BEAS-2B) was purchased from the American Type Culture Collection (ATCC). BEAS-2B cells were cultured in the DMEM supplemented with 10% FBS and 1% penicillin and streptomycin and the cells were maintained at 37 ℃ in a humidified atmosphere containing 5% CO_2_. Cell experimental group I: BEAS-2B cells were divided into five groups: control group; LPS + ATP group; LPS + ATP + HKL groups (12.5, 25, 50 μM). BEAS-2B cells in LPS + ATP + HKL groups were stimulated with LPS (1 μg/ml) for 4 h and ATP (5 mM) for 30 min [17] following 20 h pretreatment with HKL. Cell experimental group II: BEAS-2B cells were divided into four groups: control group; TBHQ (tert-Butylhydroquinone, an Nrf2 agonist) group [18]; LPS + ATP group; LPS + ATP + TBHQ (20 μM) group. BEAS-2B cells in LPS + ATP + TBHQ group were stimulated with LPS (1 μg/ml) for 4 h and ATP (5 mM) for 30 min following 20 h pretreatment with TBHQ.

### Cell counting Kit-8 (CCK-8) assay

To detect the cytotoxicity of HKL, 5 × 10^3^ cells per well of plated BEAS-2B cells were treated with 100 μl medium in 96-well plates overnight. Then, the culture medium was treated with HKL at varying concentrations (6.25, 12.5, 25, 50,100 μM). After 24 h or 48 h, 10 μl of the CCK-8 reagent were added to each well and incubated for 1.5 h away from light. The absorbance value of each well was measured on a microplate reader (Tecan Infinite F50, Switzerland) at 450 nm.

### Small interfering (si) RNA transfection

The specific human Nrf2 siRNA (5′-UCCCGUUUGUAGAUGACAA-3′) was synthesized by Ruibo Biology (Guangzhou, China). The BEAS-2B cells were cultured in the 6-well plates and transfected with siRNAs plus transfection reagent riboFECT™ CP (Guangzhou, China) according to the manufacturer’s instructions. Six hour after transfection, the culture medium was replaced. Then, the cells continued to be cultivated till 48 h. At 24 h before harvest, the cells were treated with HKL (25 μM) for 20 h, and then stimulated with LPS (1 μg/ml) for 4 h and ATP (5 mM) for 30 min.

### Animal experiments

Male SD rats (SPF) (180–220 g) were purchased from Animal Experimental Center, Tongji Medical College, Huazhong University of Science and Technology (HUST) (Wuhan, China). These rats were maintained under a controlled environment at 23 ℃ under a 12 h dark/light cycle for 1 week. The rats were randomly divided into six groups (n = 8): control group, LPS group, LPS + HKL groups (1.25, 2.5, 5 mg/kg), LPS + HKL (2.5 mg/kg) + ML385 (30 mg/kg) group. Firstly, the rats were anesthetized by injecting sodium pentobarbital (50 mg/kg) intraperitoneally, followed by subsequent intratracheal instillation of 5 mg/kg LPS in 50 μl of sterile phosphate-buffered saline (PBS) [19], while the control group was administrated with an equal volume of PBS. Half an hour after LPS administration, the rats were injected intraperitoneally with HKL (1.25, 2.5, 5 mg/kg) [20] dissolved in DMSO, while the control group was treated with PBS alone. ML385 + HKL group was treated with ML385 (30 mg/kg, i.p.)[21] before LPS administration followed by HKL (2.5 mg/kg). After LPS intervention for 24 h, the rats were euthanized by injecting 100 mg/kg sodium pentobarbital intraperitoneally. Subsequently, the bronchoalveolar lavage fluid (BALF) and lung tissues were collected.

### Specimen collection

After sacrificing the rats, the right lung was ligated, and the left lung was rinsed in 4.5 mL PBS (1.5 mL per time) to collect the BALF. To calculate the lung wet/dry ratio, the right upper lung was cut off and weighted, then was dried in an oven (60 ℃) for 72 h and weighed again. The remaining lung tissues were collected, one part was fixed in 4% paraformaldehyde for the hematoxylin and eosin (H&E) staining, and the rest was frozen at − 80 °C for extracting the RNAs and proteins.

### Western blot

The lung tissues and BEAS-2B cells were lysed with RIPA buffer with PMSF or phosphatase inhibitors. The protein concentration was detected by BCA assay kit. Equal volumes of the protein were added to each well and separated by 10% or 12% SDS-PAGE and were transferred to a PVDF (0.45 mm) membrane. The membranes were blocked in TBST containing Tween 20 (0.1%) and fat-free milk (5%) for 2 h at room temperature. The membranes were then rinsed with TBST buffer three times for 10 min and incubated with anti-NLRP3 (ABclonel, A5652, 1:500), anti-ASC (ABclonel, A1170, 1:800), anti-CASP1 (ABclonel, A0964, 1:750), anti-GSDMD (ABclonel, A18281, 1:800), anti-Nrf2 (Proteintech, 16,396–1-AP, 1:1000), anti-HO-1 (Proteintech, 10701-1-AP, 1:3000), anti-Histone H3 (Proteintech, 17168-1-AP, 1:3000), anti-GAPDH (BOSTER, BM3874, 1:1000) antibodies overnight at 4 ℃. After washing with TBST three times for 5 min, these membranes were then incubated in the rabbit HRP-conjugated secondary antibody (1:5000) at room temperature for 1 h. The bands were developed using enhanced chemiluminescence kit (NCM Biotech, P10300) and the signals were detected by a UVP BioSpectrum Imaging System (BioSpectrum600). The intensity of all bands was quantified using ImageJ software.

### Real-time polymerase chain reaction analysis (RT-PCR)

The total RNA of BEAS-2B cells and lung tissues were isolated through RNA isolation Total RNA Extraction Reagent (No: R401-01) (Vazyme, Nanjing) and then reverse-transcribed into cDNA with HiScript® II Q RT SuperMix according to the instructions. The reaction conditions were designed according to the instructions (ChamQTM SYBR®qPCR Master Mix). Thermal cycling conditions were 30 s at 95 ℃, 5 s at 95 ℃, and 30 s at 60 ℃, followed by 40 cycles, and at 95 ℃ for 15 s, 60 ℃ for 1 min, and 95 ℃ for 15 s in StepOne Plus (Applied Biosystems).The primers were synthesized by Tsingke Biology (Wuhan, China). The sequences of all primers are listed in Table 1.

**Table 1:** Sequences of primers for RT-PCR

Gene	Primer sequence	Rat (5' to 3')	Human (5' to 3')
NLRP3	Forward primer	TCTTTGCGGCTATGTACTATCT	GGACTATTTCCCCAAGATTG
	Reverse primer	TTCTAATAGGACCTTCACGT	ACTCCACCCGATGACAGTT
ASC	Forward primer	ATCCTGGACGCTCTTGAAAACTT	CTCACCGCTAACGTGCTGC
	Reverse primer	GCTCCTGTATGCCCATGTCTCTA	CTTGGCTGCCGACTGAGGA
CASP-1	Forward primer	CGGGCAAGCCAGATGTTTAT	GGGCTCTGTTTTTATTGGAA
	Reverse primer	AACCACTCGGTCCAGGAAATG	ATCTGGCTGCTCAAATGAA
GSDMD	Forward primer	CCAACATCTCAGGGCCCCAT	GGACAGGCAAAGATCGCAG
	Reverse primer	TGGCAAGTTTCTGCCCTGGA	CACTCAGCGAGTACACATTCATT
Nrf2	Forward primer	GGACCTAAAGCACAGCCAAC	TCCAGTCAGAAACCAGTGGAT
	Reverse primer	ATCTCTGGTCTGCTGCAGAG	GAATGTCTGCGCCAAAAGCTG
HO-1	Forward primer	CTTACACACCAGCCACACAG	AAGACTGCGTTCCTGCTCAAC
	Reverse primer	ACTGAGTGTGAGGACCCATC	AAAGCCCTACAGCAACTGTCG
GAPDH	Forward primer	GACATGCCGCCTGGAGAAAC	ACAACTTTGGTATCGTGGAAGG
	Reverse primer	AGCCCAGGATGCCCTTTAGT	GCCATCACGCCACAGTTTC

### Hoechst33342/Propidium Iodide (PI) staining

The cells were stained using Hoechst 33342 and PI staining solution at 4 ℃ for 20 min. After staining, the cells were rinsed once with PBS and observed under a fluorescence microscope (Olympus, Japan). The rate of PI-positive cells was analyzed using ImageJ software.

### Lactate dehydrogenase (LDH) release assay

The release of LDH in the cell supernatant was detected by the LDH kit (Jiancheng, Nanjing, China) according to the manufacturer’s instructions. The optical density (OD) value was measured at 490 nm in a microplate reader (Tecan Infinite F50, Switzerland).

### Immunofluorescence (IF) staining

BEAS-2B cells were seeded into a 24-well culture plate plated with cell climbing slices for IF at a density of 3 × 10^4^ cells/well for 12 h, then stimulated and treated as described above in the section entitled Cell culture and treatment. The cell climbing slices were washed and fixed with 4% paraformaldehyde for 20 min at room temperature, washed again and blocked with normal goat serum for 30 min at room temperature, incubated with primary antibody (GSDMD, 1:80, ABclonal, A18281) at 4 ℃ overnight, then, washed again, incubated with DyLight 488 affinipure goat anti-rabbit IgG at 37 ℃ in the dark for 1 h. Eventually, the slices were washed, protected from light and stained with DAPI for 10 min at room temperature, washed, sealed with anti-fluorescence quenching sealing tablets and observed under fluorescence microscope (IX73, Olympus, Tokyo, Japan). The immunofluorescence intensity of GSDMD was quantified using ImageJ software.

### Measurement of MPO, MDA and SOD

The activity of MPO and levels of MDA and SOD in cell supernatant and BALF were determined using the MPO, MDA and SOD assay kits (Nanjing Jiancheng Bioengineering Institute, China) according to the manufacturer’s instructions. The optical density (OD) value of MPO, MDA and SOD was measured at 460 nm, 532 nm and 450 nm in a microplate reader (Tecan Infinite F50, Switzerland).

### Histopathological analysis

The lung tissues were soaked in 10% neutral buffered formalin for 24 h and then the samples were dehydrated with graded alcohol dilutions. Subsequently, the tissues were embedded in a wax block and sliced into paraffin sections, which were stained with hematoxylin–eosin (H&E), and pathological changes of the lung tissues were observed using a light microscope (Olympus BX53 biological microscope). The lung injury was assessed independently by two blinded pathologists according to following items: neutrophils infiltration to the airspace or alveolar space, hyaline membranes formation, alveolar septal thickening, pulmonary hemorrhage [22]. The scores were as follows: no injury with a score of 0; mild to moderate injury with a score of 0.1–2.5; and severe injury with a score of 2.6–4.0 [23].

### Immunohistochemical (IHC) analysis

The lung tissues were fixed and embedded in a wax block, then sliced into 5-μm sections. The sections were dewaxed with xylene, rehydrated by fractional ethanol, repaired by antigen, and blocked with goat serum, then incubated with the primary antibody (GSDMD, 1:100, ABclonal, A18281) at 4 °C overnight, washed and incubated with secondary antibody at room temperature for 20 min, washed again and stained with DAB solution at room temperature for 6 min. Finally, the sections were washed, counterstained, dehydrated, transparentized, sealed and visualized with a light microscope (Olympus BX53 biological microscope). Image-Pro Plus 6.0 software was used to measure and analyze the intensity of IHC staining images.

### Enzyme-Linked immunosorbent assay (ELISA)

The levels of IL-1β and IL-18 in the cell supernatant and BALF were detected by ELISA. According to the manufacturer’s instructions, the working reagents were added to each well in sequence, and the optical density (OD) value was detected at 450 nm in a microplate reader (Tecan Infinite F50, Switzerland).

### Statistical analysis

All statistical data were analyzed using GraphPad Prism software v8.0. One-way ANOVA and Student’s *t*-test were used to determine the significance of the statistical results. Data are expressed as the mean ± standard deviation (SD). *P* < 0.05 was considered to be statistically significant.

## Results

### Cytotoxicity of honokiol in BEAS-2B cells

**Fig. 1:**
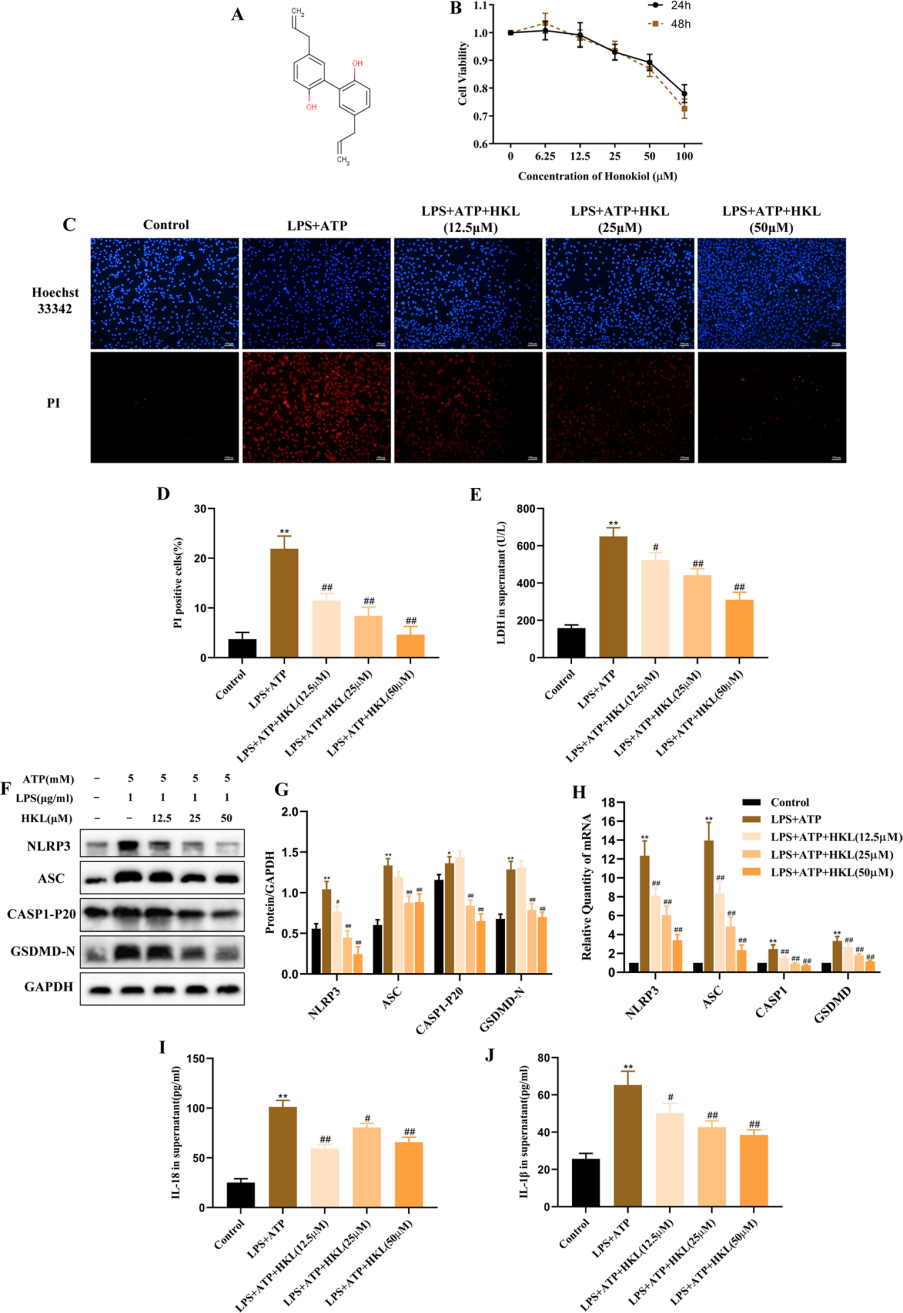
Honokiol suppresses NLRP3 inflammasome-mediated pyroptosis in BEAS-2B cells. **A** The molecular formula of honokiol (HKL). **B** BEAS-2B cells were treated with HKL (0, 6.25, 12.5, 25, 50,100 μM) for 24 or 48 h, and then cytotoxicity was detected by CCK-8 assay. BEAS-2B cells were divided into five groups: control group; LPS + ATP group; LPS + ATP + HKL groups (12.5, 25, 50 μM). BEAS-2B cells in LPS + ATP + HKL groups were stimulated with LPS (1 μg/ml) for 4 h and ATP (5 mM) for 30 min following 20 h pretreatment with HKL (**C**-**D**) using Hoechst 33,342 (blue)/PI (red) double-fluorescent staining (100 ×). Nucleus was stained using Hoechst33342. PI staining indicated the loss of plasma membrane integrity. The rate of PI-positive cells was calculated using ImageJ software. **E** LDH release in supernatant. **F**, **G** The protein expression of NLRP3, ASC, CASP1-P20, GSDMD-N were detected by western blot. **H** The mRNA expression of NLRP3, ASC, CASP1, GSDMD were detected by RT-PCR. **I**, **J** The production of IL-18 and IL-1β in supernatant were measured by ELISA. Values are expressed as the mean ± SD of three independent experiments. ^*^P < 0.05, ^**^P < 0.01 compared with the control group, ^#^*P* < 0.05, ^##^*P* < 0.01 compared with LPS + ATP group

As shown in Fig. 1A, the molecular formula of honokiol (HKL) is C_18_H_18_O_2_. To assess the cytotoxicity of HKL in BEAS-2B cells, the cells were treated with HKL (6.25, 12.5, 25, 50, 100 μM) for 24 or 48 h, then, cell viability was detected by Cell Counting Kit-8 (CCK8) assay. The results showed that the cell viability decreased to 78 and 72% after treatment with 100 μM HKL for 24 or 48 h and maintained above 80% after treatment with 50 μM HKL for 24 or 48 h (Fig. 1B). Therefore, 50 μM was determined as the highest concentration for subsequent experiments.

### Honokiol inhibits NLRP3 inflammasome-mediated pyroptosis in LPS and ATP- stimulated BEAS-2B cells

To explore the effect of HKL on pyroptosis, the BEAS-2B cells were treated with LPS and ATP, and then the level of pyroptosis was determined by calculating the ratio of PI-positive cells and the level of LDH released from the cell supernatant. Results showed that HKL significantly reduced the rate of PI-positive cells and the release of LDH in supernatant (Fig. 1C–E). Meanwhile, the markers of NLRP3 inflammasome activation, such as ASC, Caspase-1 p20 subunit (CASP1-P20), the N–terminal fragment generated from the cleavage of pyroptosis execution protein-GSDMD (GSDMD-N), IL-18 and IL-1β were detected. Additionally, HKL decreased the NLRP3, ASC, CASP1-P20 and GSDMD-N protein and NLRP3, ASC, CASP1 and GSDMD mRNA levels, and suppressed the production of Interleukin-18 (IL-18) and Interleukin-1β (IL-1β) in supernatant compared with LPS + ATP group (Fig. 1F–J). Above results suggest that HKL can prevent the pyroptosis via inhibiting NLRP3 inflammasome activation and GSDMD cleavage in BEAS-2B cells induced by LPS and ATP.

### Honokiol activates Nrf2 and improves the oxidative stress in LPS and ATP-stimulated BEAS-2B cells

**Fig. 2:**
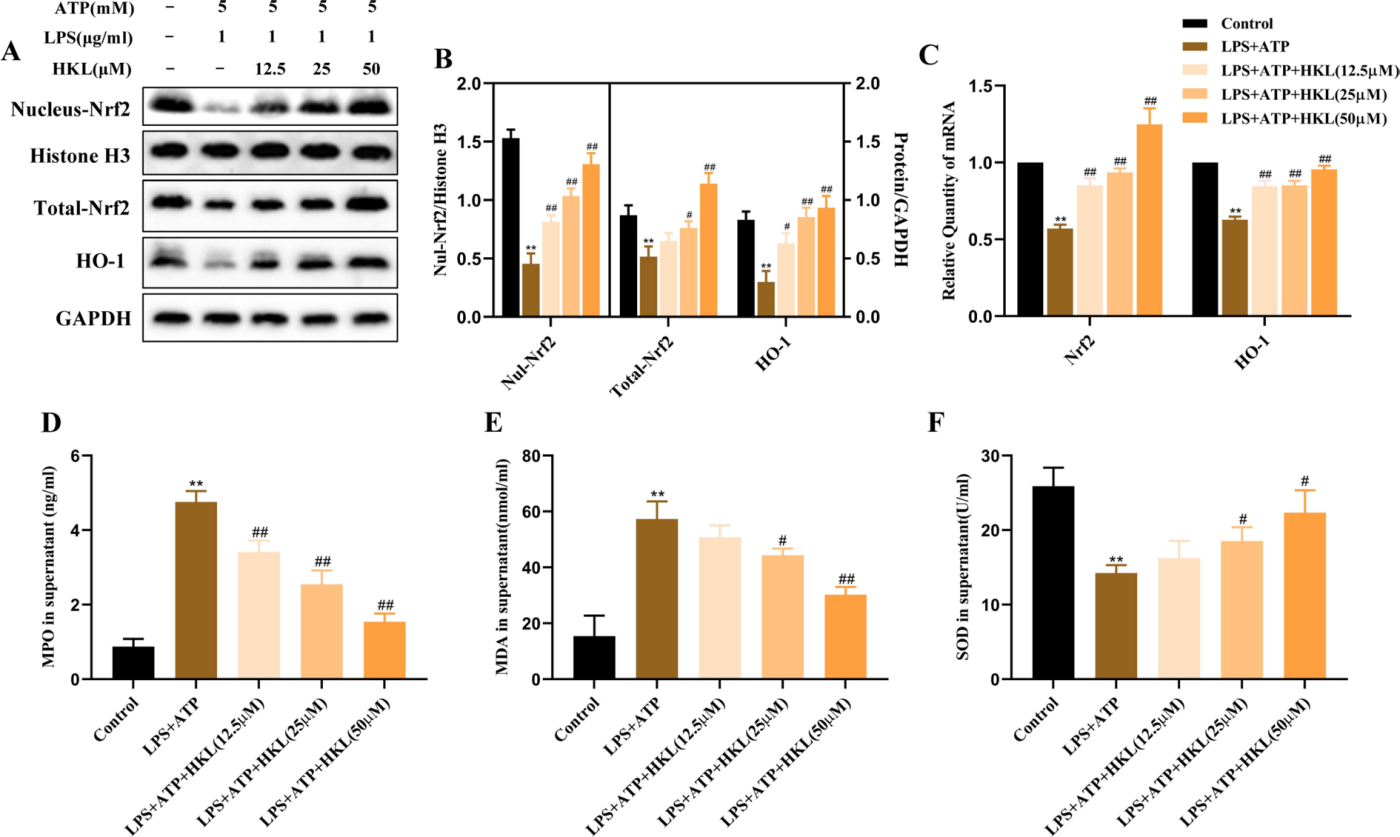
Honokiol activates Nrf2/HO-1 and improves the oxidative stress in BEAS-2B cells. BEAS-2B cells were stimulated with LPS (1 μg/ml) for 4 h and ATP (5 mM) for 30 min in the presence or absence of HKL. **A**, **B** The protein expression of Total-Nrf2, Nul-Nrf2 and HO-1 was measured by western blot. **C** The mRNA expression of Nrf2 and HO-1 was measured by RT-PCR. **D**–**F** The levels of MPO, MDA and SOD in supernatant were measured by ELISA. Values are expressed as the mean ± SD of three independent experiments. ^*^P < 0.05, ^**^P < 0.01 compared with the control group, ^#^*P* < 0.05, ^##^*P* < 0.01 compared with LPS + ATP group

HKL was recently reported to possess strong anti-oxidative activity [24], however, the anti-oxidant effect of HKL in lung epithelial cells has not been studied. Thus, we explored the anti-oxidant effect and potential mechanism of HKL on BEAS-2B cells after LPS and ATP stimulation. Firstly, we evaluated the effect of HKL on oxidative stress markers such as MDA, MPO, and SOD, and found that HKL significantly reduced MPO and MDA levels, and increased SOD levels in BEAS-2B cells (Fig. 2D–F). To explore the anti-oxidative mechanism of HKL in BEAS-2B cells, we assessed the effect of HKL on Nrf2. Nrf2, as a transcription factor, can regulate oxidative stress [25]. The results showed that HKL could promote Nrf2 translocation into the nucleus, and increase the protein and mRNA expression of total Nrf2 and HO-1 (Fig. 2A–C). These results altogether indicated that HKL could reduce the oxidative stress and activate Nrf2.

### Honokiol exerts its anti-oxidative effect in LPS and ATP-stimulated BEAS-2B cells via activation of Nrf2

**Fig. 3:**
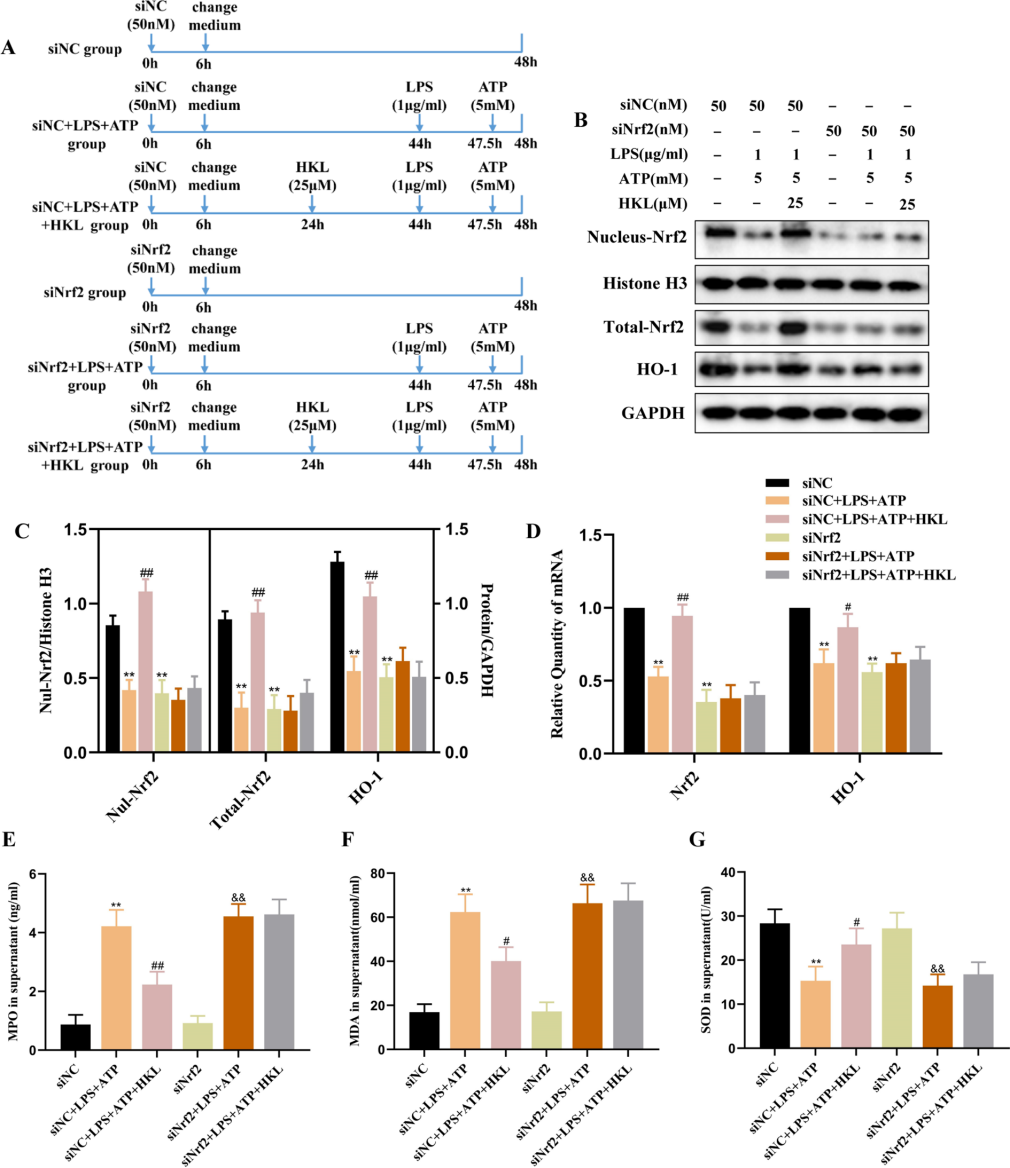
The antioxidant effect of HKL in BEAS-2B cells depends on the activation of Nrf2. After transfection with negative control siRNA (siNC) or Nrf2 siRNA (siNrf2) for 6 h, the cell medium was changed and BEAS-2B cells continued to be cultivated for up to 48 h. At 24 h before harvest, the cells were treated with HKL (25 μM) for 20 h, and then stimulated with LPS (1 μg/ml) for 4 h and ATP (5 mM) for 30 min. **A** Flow diagrams of cell experiments. **B**, **C** The protein expression of Total-Nrf2, Nul-Nrf2 and HO-1 and **D** mRNA expression of Nrf2 and HO-1 were analyzed by western blot and RT-PCR. **E**–**G** The levels of MPO, MDA and SOD in supernatant were quantified by ELISA. Values are expressed as the mean ± SD of three independent experiments. ^**^P < 0.01 compared with the siNC group; ^#^*P* < 0.05, ^##^*P* < 0.01 compared with siNC + LPS + ATP group; ^&^*P* < 0.05, ^&&^*P* < 0.01 compared with siNrf2 group

To verify whether the anti-oxidant effect of HKL in LPS and ATP-stimulated BEAS-2B cells is related to Nrf2, Nrf2 was knocked down by the siRNA technology (Fig. 3A) and found that the regulation of HKL on Nrf2, HO-1, MPO, MDA and SOD in LPS and ATP-stimulated BEAS-2B cells were reversed (Fig. 3B–G), which suggested that the anti-oxidative property of HKL in LPS and ATP-stimulated BEAS-2B cells was at least partially dependent on the activation of Nrf2.

### Activation of Nrf2 inhibits NLRP3 inflammasome-mediated pyroptosis in BEAS-2B cells

**Fig. 4:**
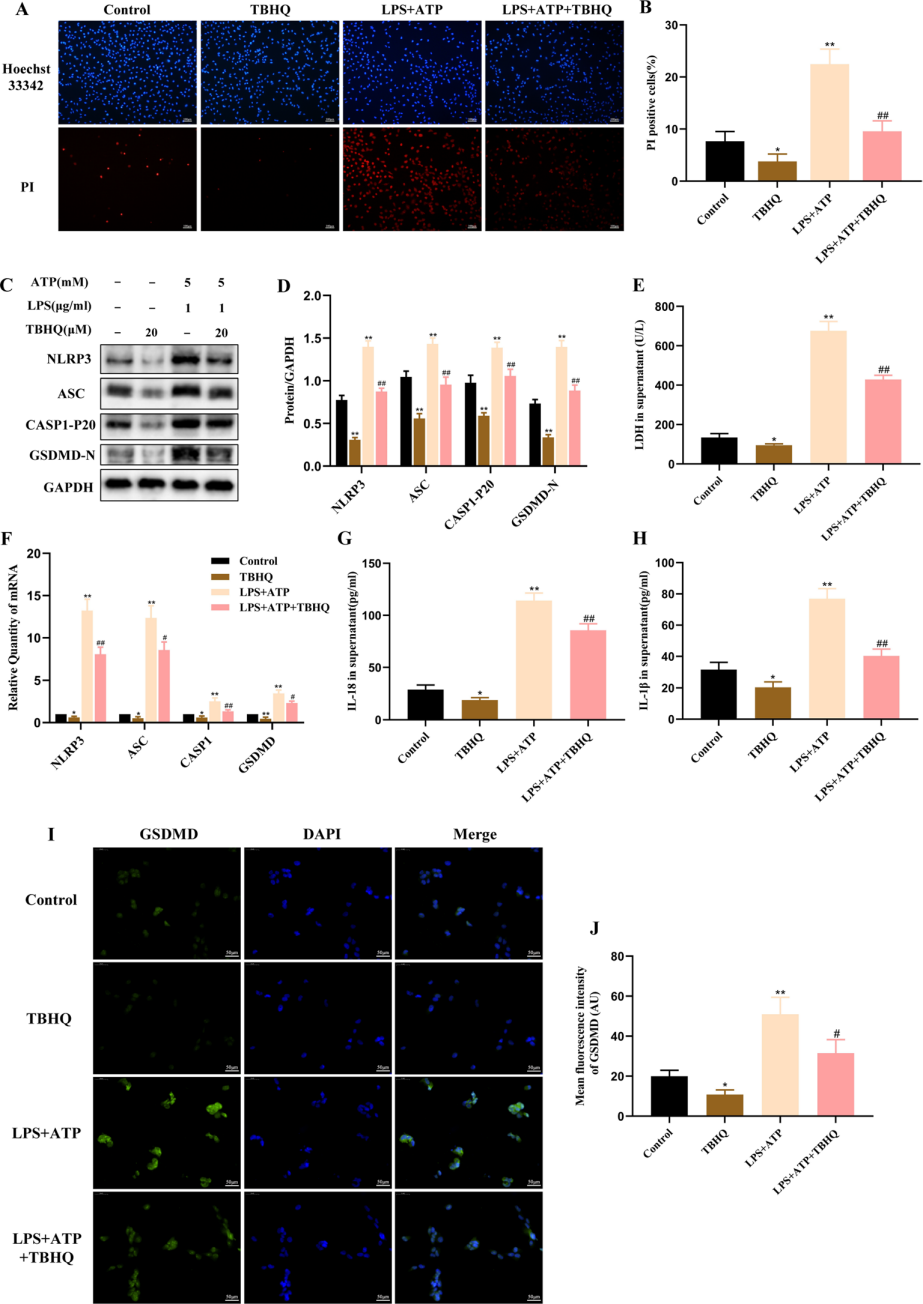
Activation of Nrf2 inhibits NLRP3 inflammasome-mediated pyroptosis in BEAS-2B cells. BEAS-2B cells were divided into four groups: control group; TBHQ group; LPS + ATP group; LPS + ATP + TBHQ (20 μM) group. BEAS-2B cells in LPS + ATP + TBHQ group were stimulated with LPS (1 μg/ml) for 4 h and ATP (5 mM) for 30 min following 20 h pretreatment with TBHQ. **A**, **B** Hoechst 33,342 (blue)/PI (red) double-fluorescent staining (100 ×) and the rate of PI-positive cell. **C**, **D** The protein expression of NLRP3, ASC, CASP1-P20, GSDMD-N were detected by western blot. **E** LDH release in supernatant. **F** The mRNA expression of NLRP3, ASC, CASP1, GSDMD were detected by RT-PCR. **G**, **H** The production of IL-18 and IL-1β in supernatant were measured by ELISA. **I** Immunofluorescence staining of GSDMD (original magnification 400 ×). Merged images of DAPI for nucleus (blue) and GSDMD immunofluorescence (green). **J** The mean fluorescence intensity (MFI) was quantified using ImageJ software. MFI = Integrated Density/Area. Values are expressed as the mean ± SD of three independent experiments. ^*^P < 0.05, ^**^P < 0.01 compared with the control group, ^#^P < 0.05, ^##^P < 0.01 compared with LPS + ATP group

Nrf2 has been recently shown to be the key factor in the activation of the NLRP3 inflammasome [26]. However, it is not clear whether NRF2 can regulate NLRP3 inflammasome-mediated pyroptosis in BEAS-2B cells stimulated with LPS and ATP. So we pretreated BEAS-2B cells with TBHQ (an Nrf2 agonist) to observe whether it affects pyroptosis. As shown in Fig. 4A–J, the rate of PI-positive cells, release of LDH, protein expression of NLRP3, ASC, CASP1-P20, GSDMD-N, mRNA expression of NLRP3, ASC, CASP1, GSDMD, production of IL-18 and IL-1β in THBQ group were totally decreased than those in the control groups. Meanwhile, the levels of these pyroptosis-related indexes in TBHQ pretreatment group were also significantly lower than those in LPS + ATP group. The above results showed that activation of Nrf2 could inhibit the NLRP3 inflammasome-mediated pyroptosis in BEAS-2B cells stimulated with LPS and ATP.

### Honokiol suppresses NLRP3 inflammasome-mediated pyroptosis in LPS and ATP stimulated-BEAS-2B cells via activation of Nrf2

**Fig. 5:**
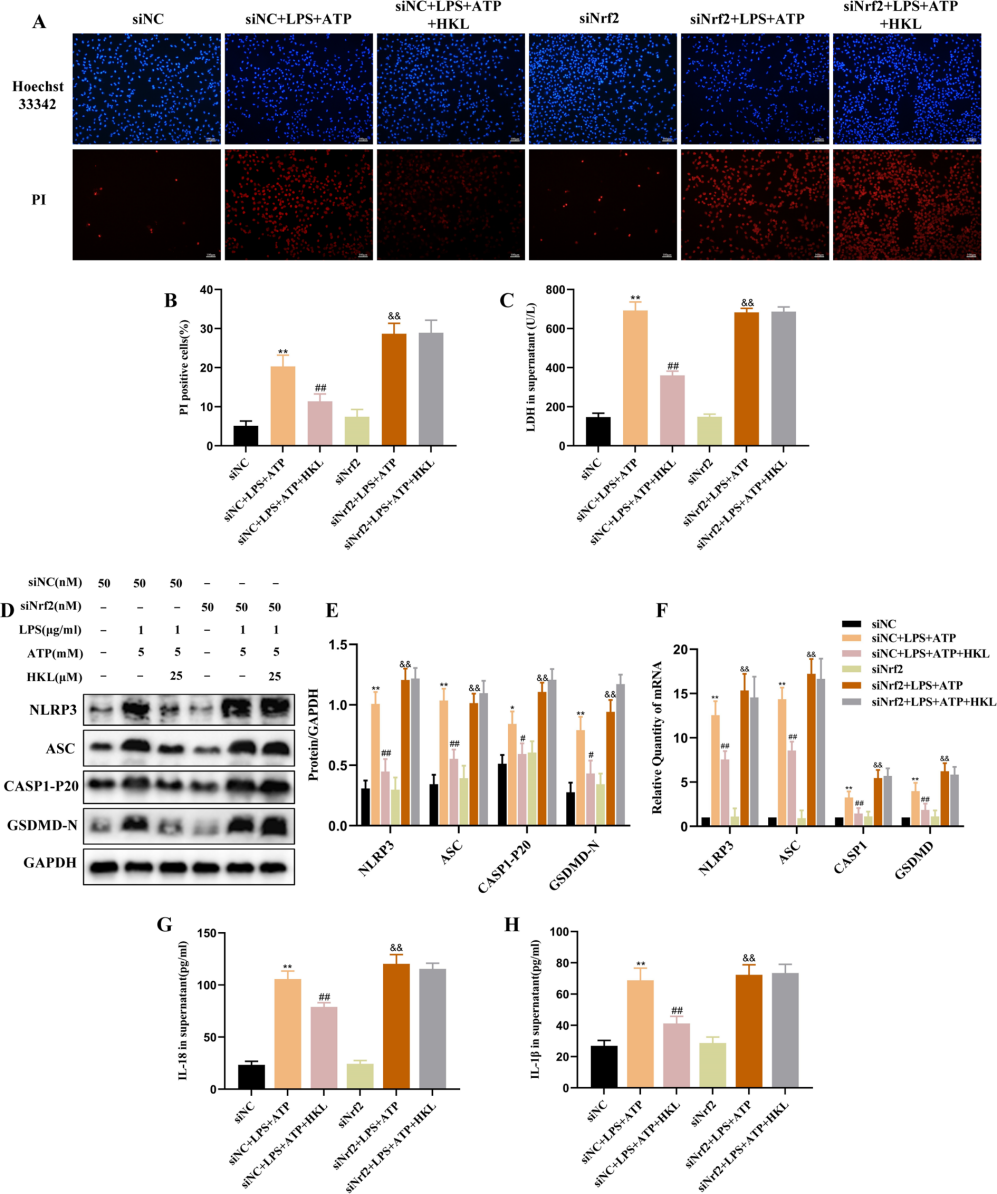
Honokiol inhibits NLRP3 inflammasome-mediated pyroptosis through activation of Nrf2 in BEAS-2B cells. **A**, **B** Hoechst 33,342 /PI double-fluorescent staining was used to assess the pyroptosis of LPS and ATP-stimulated BEAS-2B cells (original magnification × 100). **C** The content of LDH in supernatant was measured by an LDH assay kit. **D**, **E **The protein expression of NLRP3, ASC, CASP1-P20, GSDMD-N and (F) mRNA expression of NLRP3, ASC, CASP1, GSDMD were analyzed by western blot and RT-PCR. **G**, **H** The production of IL-18 and IL-1β in supernatant were measured by ELISA. Values are expressed as the mean ± SD of three independent experiments. ^**^P < 0.01 compared with the siNC group; ^#^*P* < 0.05, ^##^*P* < 0.01 compared with siNC + LPS + ATP group; ^&^*P* < 0.05, ^&&^*P* < 0.01 compared with siNrf2 group

To observe whether the inhibitory effect of HKL on NLRP3 inflammasome-mediated pyroptosis was linked to the activation of Nrf2, we knocked down Nrf2 in BEAS-2B cells using siRNA technology. The results suggested that after siRNA-NC transfection, HKL could still inhibit the protein expression of NLRP3, ASC, CASP1-P20, GSDMD-N protein and mRNA expression of NLRP3, ASC, CASP1, GSDMD, decrease the rate of PI-positive cells positive and the release of IL18, IL-1β and LDH in cell supernatant (Fig. 5A–H). However, after siRNA-Nrf2 administration, the inhibition of HKL on NLRP3 inflammasome-mediated pyroptosis was significantly reversed (Fig. 5A–H). Thus, the inhibitory effect of HKL on the NLRP3 inflammasome-mediated pyroptosis was discovered to be partially dependent on the activation of Nrf2.

### Honokiol attenuates LPS-induced ALI in rats

**Fig. 6:**
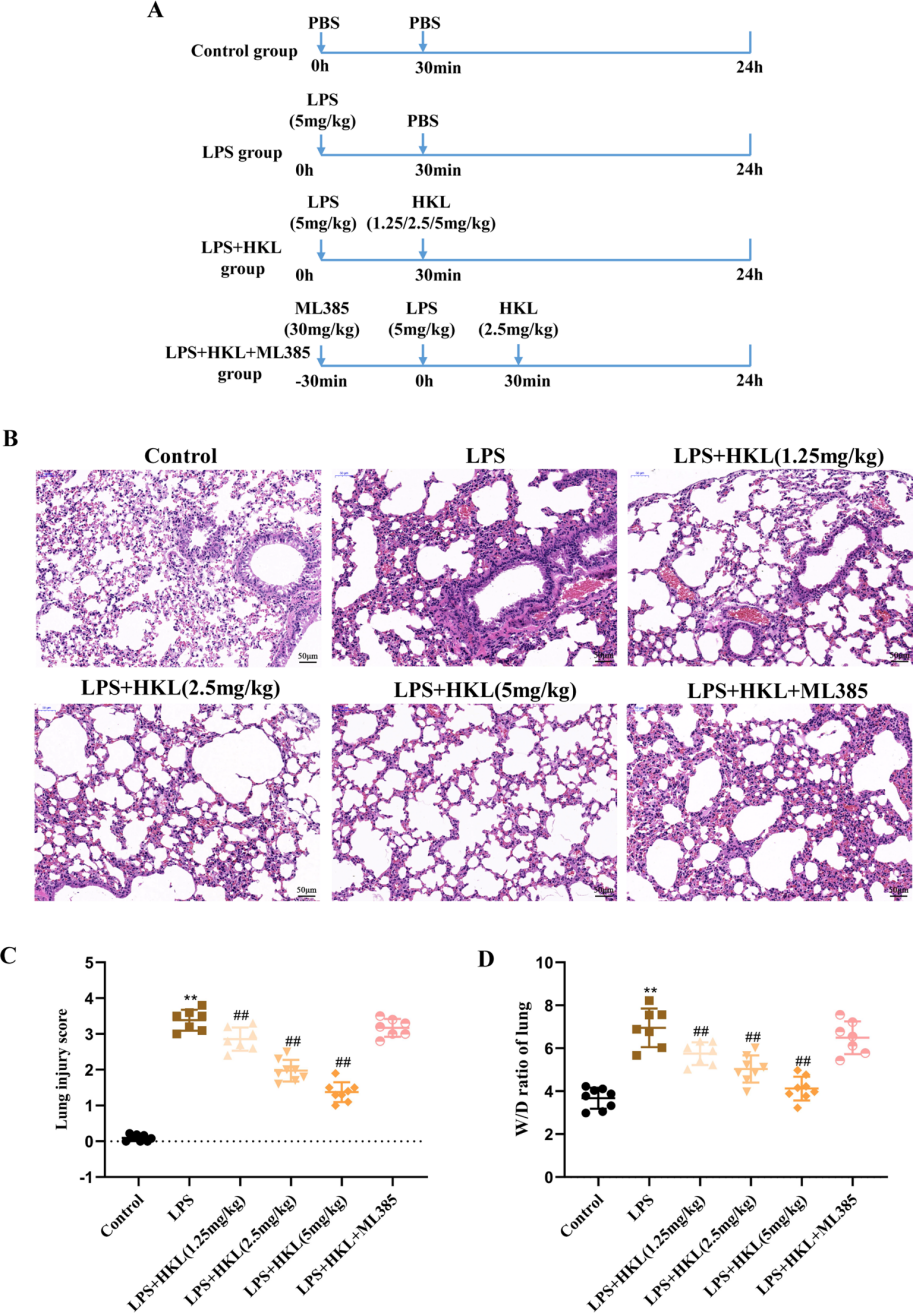
Honokiol attenuates LPS-induced ALI. The male SD rats were intratracheally instilled with LPS (5 mg/kg) to establish the ALI model. Then rats were administrated with HKL (1.25, 2.5, 5 mg/kg) or ML385 (30 mg/kg) intraperitoneally based on the grouped information. Rats were euthanized 24 h after LPS administration. **A** Flow diagrams of animal experiments, the rats were divided into six groups: control group, LPS group, LPS + HKL (1.25, 2.5, 5 mg/kg) groups, LPS + HKL (2.5 mg/kg) + ML385 (30 mg/kg) group. **B** Representative images of H&E staining (original magnification × 200). **C** Lung injury score. **D** Lung wet/dry weight ratio. Data were analyzed using one-way ANOVA; n = 7–8 rats per group. ^**^P < 0.01 compared with the control group; ^#^*P* < 0.05, ^##^*P* < 0.01 compared with LPS group

To determine the protective effect of HKL on LPS-induced ALI rats, the pathological changes of rat lung tissues were assessed by H&E staining. As shown in Fig. 6B, C, The ALI scores of LPS group were significantly higher than the control group and the pathological changes were characterized by increased accumulation of inflammatory cells, alveolar hemorrhage and pulmonary interstitial edema. However, the degree of pathological injury of lung tissues and the ALI scores in LPS + HKL groups were significantly improved and decreased. At the same time, the lung wet/dry (W/D) weight ratio was detected to evaluate the edema of rat lung tissues. As shown in Fig. 6D, the lung W/D weight ratio of the rats in the LPS group was significantly increased compared with the control group, while HKL treatment could significantly reduce the ratio. The results suggested that HKL could effectively attenuate LPS-induced ALI in rats. Interestingly, it was found that pretreatment with ML385 could significantly weaken the protective effect of HKL on LPS-induced ALI, which suggested that the protective effect of HKL on LPS-induced ALI was at least partially related to the activation of Nrf2.

### Honokiol exerts an anti-oxidative effect to attenuate LPS-induced ALI via activation of Nrf2

**Figure7:**
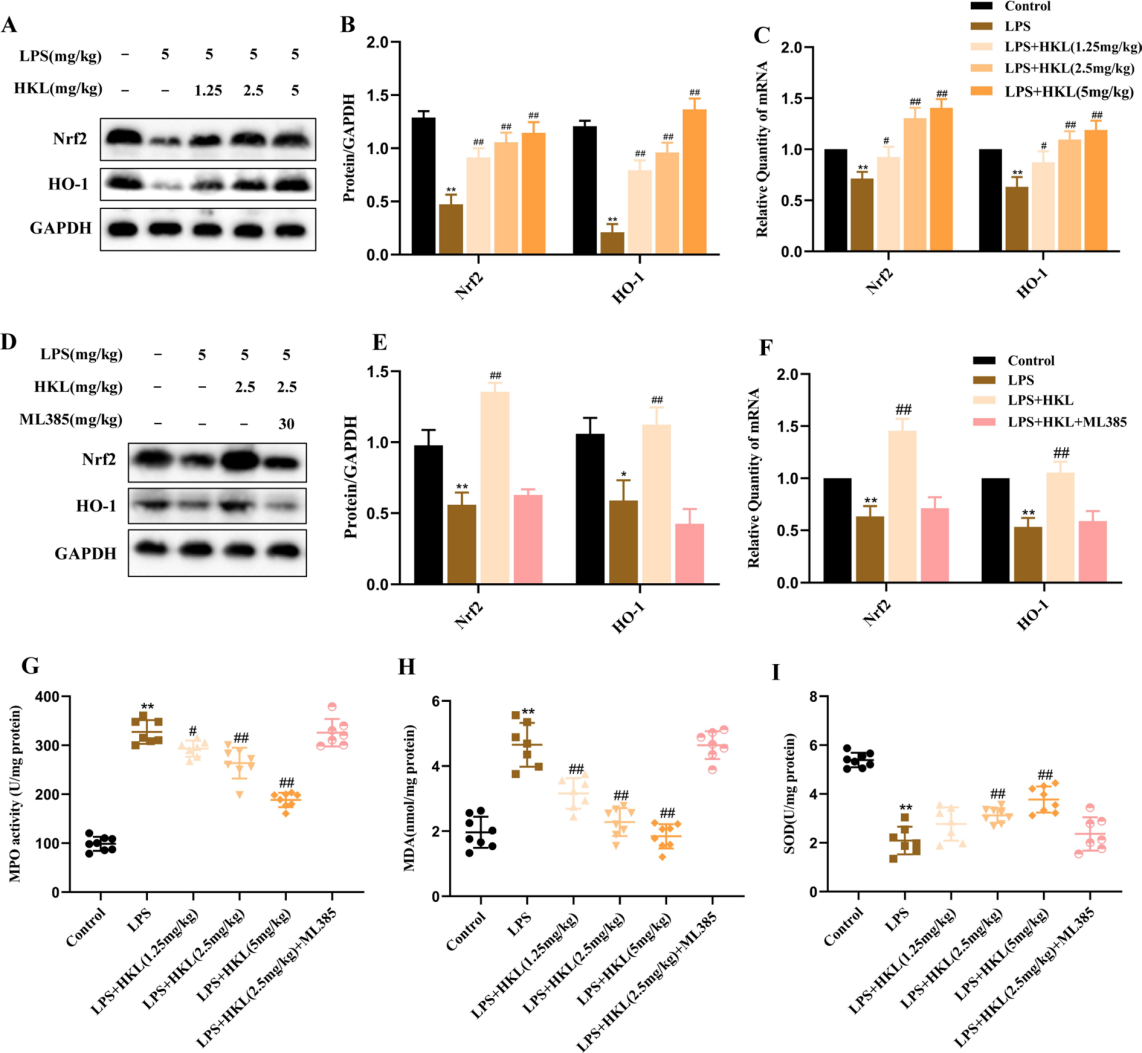
Honokiol attenuates the oxidative stress in rats with ALI via activation of Nrf2. **A**, **B**, **D**, **E** Relative expression levels of Nrf2 and HO-1 protein in lung tissues analyzed by Western blot. **C**, **F** Relative expression levels of Nrf2 and HO-1 mRNA in lung tissues were analyzed by RT-PCR. **G**–**I** The levels of MPO, MDA and SOD in bronchoalveolar lavage fluid of each group were detected by ELISA. Data were analyzed using one-way ANOVA; n = 7–8 rats per group. ^**^P < 0.01 compared with the control group; ^#^*P* < 0.05, ^##^*P* < 0.01 compared with LPS group

To investigate whether the protective mechanism of HKL against LPS-induced ALI is related to its anti-oxidant activity, the activities of the oxidative stress markers, such as MPO, MDA, SOD were determined, and the Nrf2 and HO-1 expression levels were also detected. It was found that LPS administration could significantly increase the activities of MPO and MDA, and inhibit the level of SOD in BALF, but, all of which could be reversed by pretreatment of HKL (Fig. 7G–I). Additionally, we observed that HKL increased the protein and mRNA level of Nrf2 and HO-1 in rat lung tissues (Fig. 7A–C). To further verify whether the anti-oxidative protection of HKL on LPS-induced ALI depended on the activation of Nrf2, the rats were pretreated with ML385, an inhibitor of Nrf2. Interestingly, the regulatory effect of HKL on Nrf2, HO-1, and oxidative stress markers was significantly blocked by ML385 (Fig. 7D–I). Accordingly, we proposed that the anti-oxidative protection of HKL on LPS-induced ALI was dependent on Nrf2 activation.

### Honokiol inhibits NLRP3 inflammasome-mediated pyroptosis in LPS induced-ALI rats via activation of Nrf2

To investigate the anti-inflammatory and protective mechanisms of HKL on LPS-induced ALI, we observed whether HKL could inhibit NLRP3 inflammasome-mediated pyroptosis in vivo.

**Fig. 8:**
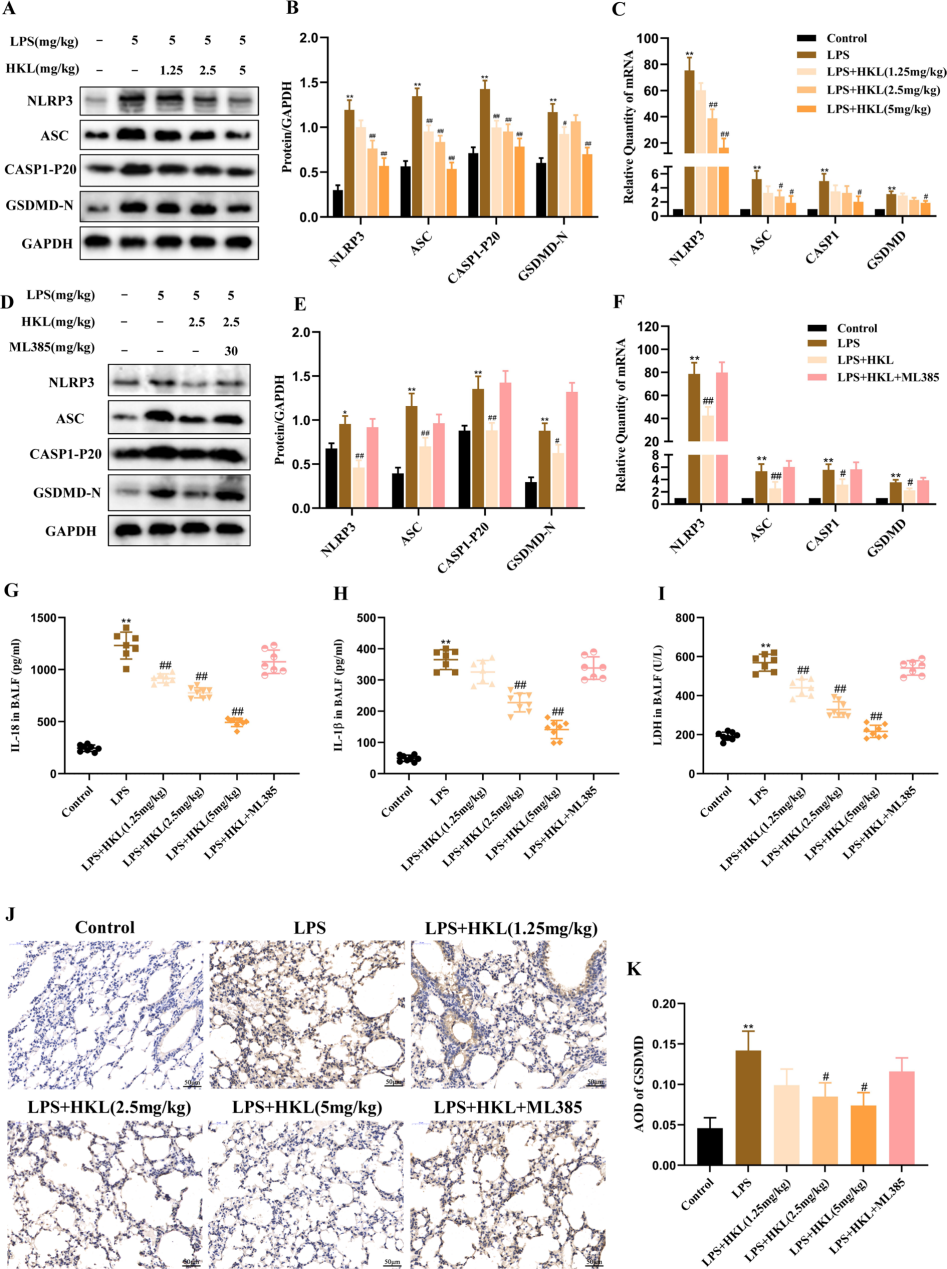
Honokiol inhibits NLRP3 inflammasome-mediated pyroptosis in rats with ALI via activation of Nrf2. **A**–**B**, **D**–**E** Relative expression levels of NLRP3, ASC, CASP1-P20, GSDMD-N protein in lung tissues were analyzed by western blot. **C**, **F** Relative expression levels of NLRP3, ASC, CASP1, GSDMD mRNA in lung tissues were analyzed by RT-PCR. **G**–**I** The content of IL-18, IL-1β and LDH in bronchoalveolar lavage fluid of each group were detected by ELISA. **J** Immunohistochemical staining of GSDMD (original magnification 400 ×) in the rat lungs. **K** The average optical density (AOD) was used to evaluate the intensity of IHC staining and measured by Image-Pro Plus 6.0 software. Data were analyzed using one-way ANOVA; n = 7–8 rats per group. ^**^P < 0.01 compared with the control group; ^#^*P* < 0.05, ^##^*P* < 0.01 compared with LPS group

The results indicated that after LPS stimulation, the protein levels of NLRP3, ASC, CASP1-P20 and GSDMD-N and mRNA levels of NLRP3, ASC, CASP1 and GSDMD in lung tissues and the release of LDH, IL-18, IL-1β in BALF were significantly increased (Fig. 8A–C, G–K). Consistent with the results in vitro, HKL was found to suppress the activation of NLRP3 inflammasome and reduce the release of LDH, IL-18 and IL-1β (Fig. 8A–C, G–K). To verify whether the inhibitory effect of HKL on NLRP3 inflammasome-mediated pyroptosis in vivo is related to the activation of Nrf2, the rats were pretreated with ML385, an Nrf2 inhibitor. After the administration of ML385, HKL could neither downregulate the NLRP3, ASC, CASP1-P20, GSDMD-N protein levels and NLRP3, ASC, CASP1, GSDMD mRNA levels of in the lung tissues, nor reduce the release of IL18, IL1β and LDH in the BALF (Fig. 8D–K). To summarize the above results, HKL was suggested to exert its anti-inflammatory effect to attenuate the LPS-induced ALI via inhibiting the NLRP3 inflammasome-mediated pyroptosis, which was at least partially dependent on the activation of Nrf2.

## Discussion

ALI and ARDS constitute serious respiratory diseases with high morbidity and mortality [27]. However, there is no specific treatment for ALI to date. With the development of phytochemistry and pharmacology research, a large number of natural products with anti-oxidant, anti-inflammatory and anti-bacterial activities have been found. Therefore, there is an increasing demand for the botanical components of traditional medicine as a source of prevention and treatment for ALI [28–30]. HKL, a natural compound extracted from Magnolia Officinalis, has been reported to have anti-oxidant and anti-inflammatory effects. This study aimed to investigate the therapeutic effects and protective mechanisms of HKL on ALI induced by LPS.

The pathogenesis of ALI is still not conclusively known. Lung barrier damage, lung epithelial injury, inflammation and oxidative damage are the main characteristics of ALI [31–34]. Accumulating pieces of evidence suggest that airway epithelium is involved in the pathogenesis of ALI / ARDS [35, 36]. The airway epithelium is the body’s first line of defense against lung injury [4]. Previous studies have shown that the activation of airway epithelial cells could produce pro-inflammatory factors, which aggravated the development of pulmonary edema in LPS induced-ALI [3]. It has been reported that excessive activation of NLRP3 inflammasome aggravated lung inflammation and tissue damage [37]. Activation of NLRP3 inflammasome could promote the formation of GSDMD pore and the secretion of IL-1β, IL-18 via CASP1 activation, thereby inducing the occurrence of pyroptosis [38–41]. However, the role of NLRP3 inflammasome-mediated pyroptosis in airway epithelial cells for ALI has not been studied.

In our study, in vitro, BEAS-2B cells were stimulated with LPS and ATP to induce pyroptosis, and the rate of PI-positive cells, the release of LDH, IL-18, IL-1β, and the NLRP3, ASC, CASP1-P20, GSDMD-N protein levels and NLRP3, ASC, CASP1, GSDMD mRNA levels were significantly increased. Meanwhile, HKL was found to reverse the changes of the above indicators. In addition, the anti-pyroptotic effect of HKL has also been demonstrated in vivo. The rat model of ALI was established by intratracheal instillation of LPS. It was found that HKL could decrease the protein levels of NLRP3, ASC, CASP1-P20, GSDMD-N and mRNA levels of NLRP3, ASC, CASP1, GSDMD in lung tissues, and the content of LDH, IL-18, IL-1β in BALF. The above results suggest that HKL may improve LPS-induced ALI by inhibiting NLRP3 inflammasome-mediated pyroptosis in airway epithelial cells, and also indicate that pyroptosis in airway epithelium may be one of the potential targets for the prevention and treatment of ALI.

Disorders associated with oxidative stress can increase lung inflammation and aggravate the progression of ALI [42, 43]. Nrf2, as a basic leucine zipper redox-sensitive transcription factor, can regulate oxidative stress and inflammation [44, 45]. Activation of Nrf2 can facilitate the expression of HO-1, defending against oxidative stress [46, 47]. Recent research showed that the activation of Nrf2/HO-1 signaling pathway could protect the oxidative stress-related damage in ALI [48, 49]. As previously mentioned, HKL has strong antioxidant activity, so we hypothesized that HKL could reduce oxidative stress and attenuate LPS-induced ALI via activation of Nrf2/HO-1 signaling pathway. Our results suggested that HKL could significantly upregulate the protein and mRNA expression levels of Nrf2 and HO-1, decrease the levels of MPO, MDA, and enhance the level of SOD in vivo and in vitro. And we also discovered that the anti-oxidant effect of HKL could be reversed by Nrf2 knockdown, which indicated that HKL played an anti-oxidative role in LPS-induced ALI via regulating Nrf2/HO-1 signaling pathway.

The current study also showed that Nrf2 negatively regulated the activation of NLRP3 inflammasome [50, 51]. It has been reported that Nrf2 could inhibit the activation of NLRP3 inflammasome by enhancing Trx activity and suppressing TXNIP in human-derived neuronal cells [52], and Nrf2-antioxidant signaling could inhibit the NLRP3/IL-1β pathway in human corneal epithelial cells [53]. Meanwhile, studies have revealed that Nrf2/HO-1 pathway could suppress NF-κB-p65 phosphorylation to inhibit NLRP3 inflammasome activation in myeloid-derived suppressor cells [54]. In addition, ROS-induced NLRP3 inflammasome activation in BV2 cells was also related to Nrf2 / ARE pathway [55]. However, it is not clear whether Nrf2 can regulate NLRP3 inflammasome activation in BEAS-2B cells. Therefore, we pretreated the cells with TBHQ (an Nrf2 agonist) and observed its effect on NLRP3 inflammasome activation. The results showed that TBHQ could significantly inhibit the activation of NLRP3 inflammasome and reduce the degree of pyroptosis. Our preceding results suggest that HKL activates Nrf2 and inhibits NLRP3 inflammasome-mediated pyroptosis, then, we advanced the conjecture that the inhibitory effect of HKL on NLRP3 inflammasome-mediated pyroptosis may be due to the Nrf2 activation. To verify this conjecture, siRNA was used to silence Nrf2 in vitro and ML385 (an Nrf2 inhibitor) was used to block the activation of Nrf2 in vivo. Interestingly, we found that after the Nrf2 knockdown, the inhibitory effect of HKL on NLRP3 inflammasome-mediated pyroptosis was reversed in vitro and in vivo. These results suggest that HKL may inhibit NLRP3 inflammasome-mediated pyroptosis by activating Nrf2, which plays a protective role in LPS-induced ALI.

## Conclusion

In this study, we found that HKL could attenuate NLRP3 inflammasome-mediated pyroptosis in BEAS-2B cells via activating Nrf2. Meanwhile, we verified that HKL could inhibit NLRP3 inflammasome-mediated pyroptosis by activating Nrf2 to reduce LPS-induced ALI. Overall, these results reveal the new protective mechanism of HKL on ALI, providing an experimental basis for their clinical applications in the future. However, numerous safety and efficacy studies are necessary before its clinical applications. Furthermore, the specific mechanism of the connection between Nrf2 and NLRP3 inflammasome needs to be further explored in future studies.

## Data Availability

The data analyzed during this study can be obtained from the corresponding author on reasonable request.

## Abbreviations

HKL: Honokiol
LPS: Lipopolysaccharide
ALI: Acute lung injury
NLRP3: NOD-like receptor family pyrin domain containing 3
ASC: Apoptosis-associated speck-like protein containing a caspase-recruitment domain
CASP1: Caspase-1
GSDMD: Gasdermin D
IL-1β: Interleukin-1beta
Nrf2: Nuclear factor erythroid-2 related factor 2
HO-1: Heme oxygenase-1
MPO: Myeloperoxidase
MDA: Malondialdehyde
SOD: Superoxide dismutase
PI: Propidium iodide
BEAS-2B: Human bronchial epithelial cell line
PBS: Phosphate buffer saline
DMEM: Dulbecco’s modified Eagle’s medium
FBS: Fetal bovine serum
siRNA: Small interfering RNA
BALF: Bronchoalveolar lavage fluid
PMSF: Phenylmethanesulphonyl fluoride
RIPA: RIPA Lysis Buffer
CCK8: Cell counting Kit-8
LDH: Lactate dehydrogenase
i.p.: Intraperitoneal
H&E: Hematoxylin and eosin
